# Dynamic changes in fractional amplitude of low-frequency fluctuations in patients with chronic insomnia

**DOI:** 10.3389/fnins.2022.1050240

**Published:** 2022-11-29

**Authors:** Wei Chen, Hui Wang, Tianze Sun, Qi Wu, Wenxuan Han, Qian Li, Yong Liu, Yuanping Zhou, Xiuyong He

**Affiliations:** ^1^Department of Medical Imaging Center, Ankang Hospital of Traditional Chinese Medicine, Ankang, China; ^2^School of Future Technology, Xi’an Jiaotong University, Xi’an, China; ^3^Department of Medical Imaging, The First Affiliated Hospital of Xi ‘an Jiaotong University, Xi’an, China

**Keywords:** dynamic fractional amplitude of low-frequency fluctuations, chronic insomnia, sleep disorder, multivariate pattern analysis, instable brain activity

## Abstract

**Background:**

Previous neuroimaging studies have mostly focused on changes in static functional connectivity in patients with ***chronic insomnia (CI)***. Features of dynamic brain activity in patients with CI have rarely been described in detail. The present study investigated changes in dynamic intrinsic brain activity in patients with CI by dynamic fractional amplitude of low-frequency fluctuation (dfALFF) analysis.

**Materials and methods:**

A total of 30 patients with CI and 27 healthy controls (HCs) were enrolled. We compared dfALFF between these two groups, and examined the correlation between changes in dfALFF and clinical symptoms of CI. Multivariate pattern analysis was performed to differentiate patients with CI from HCs.

**Results:**

Compared with HC subjects, patients with CI showed significantly increased dfALFF in the left insula, right superior temporal gyrus, left parahippocampal gyrus, right amygdala, and bilateral posterior lobes of the cerebellum. Moreover, dfALFF values in the left insula and left parahippocampal gyrus showed a positive correlation with Pittsburgh Sleep Quality Index scores. A logistic regression model was constructed that had 96.7% sensitivity, 80.0% specificity, and 83.0% overall accuracy for distinguishing patients with CI from HCs.

**Conclusion:**

Dynamic local brain activity showed increased instability in patients with CI. The variability in dfALFF in the limbic system and brain areas related to sleep/wakefulness was associated with insomnia symptoms. These findings may provide insight into the neuropathologic basis of CI.

## Introduction

The Diagnostic and Statistical Manual of Mental Disorders, Fifth Edition (DSM-V) defines chronic insomnia (CI) as dissatisfaction with sleep quality or quantity, characterized by one or more of the following symptoms lasting for over 3 months: (1) Difficulty in initiating sleep; (2) difficulty in maintaining sleep; and (3) early awakening and inability to return to sleep. Insomnia is a widespread and serious problem that affects the global population (Morin et al., 2006); the combination of insomnia and physical exhaustion can lead to attention deficit and memory loss, which can greatly affect patients’ work productivity and ability to learn (de Zambotti et al., 2018; Gierc et al., 2022). Long-term sequelae of insomnia include endocrine disorders, hypertension, and other diseases (Khan and Aouad, 2022). Insomnia can also increase the probability of depression and anxiety disorders as well as suicide risk (Tucker et al., 2021). However, the neurobiologic mechanisms of insomnia are not fully understood.

Advances in neuroimaging technologies have facilitated the investigation of neural mechanisms underlying insomnia (Tahmasian et al., 2018; Fasiello et al., 2022). Neuroanatomical studies have revealed abnormal structural changes in the orbitofrontal cortex (Stoffers et al., 2012; Xie et al., 2020), cingulate cortex (Li et al., 2018), insula (Yu et al., 2020), amygdala (Koo et al., 2017; Emamian et al., 2021), and cerebellum (Joo et al., 2013). Functional magnetic resonance imaging (fMRI) has been widely used to explore changes in brain activation in insomnia (Jiang et al., 2020; Fasiello et al., 2022). One fMRI study found that insomnia patients had weakened functional connectivity primarily in the right dorsolateral prefrontal cortex, left insula, and right cerebellum anterior lobe (Huang et al., 2017). Regional homogeneity (ReHo) and amplitude of low-frequency fluctuation (ALFF) are the most frequently used and reliable parameters for evaluating local spontaneous neural activity (Zang et al., 2004, 2007). Fractional ALFF (fALFF) reduces physiologic noise and improves the accuracy of ALFF measurements (Zou et al., 2008). Abnormal ReHo and ALFF have been observed in specific brain regions of patients with insomnia including the fusiform gyrus, prefrontal cortex, insula, cingulate gyrus, and cerebellum (Dai et al., 2014; Wang et al., 2016; Pang et al., 2018); and alterations in ALFF were shown to be correlated with sleep quality and psychological performance (Zhou et al., 2017; Zhang et al., 2021). Most of these studies were based on the assumption that brain activity is static during resting-state fMRI scanning, but there is increasing evidence that brain activity and function are temporally dynamic (Hutchison et al., 2013; Allen et al., 2014; Cui et al., 2020). A dynamic ALFF (dALFF) method involving a sliding window technique to calculate changes in ALFF over time (Fu et al., 2018; Liao et al., 2019) has been applied to a variety of neurologic diseases and mental disorders such as mild cognitive impairment (Wang et al., 2019), Parkinson’s disease (Tian et al., 2022), obsessive-compulsive disorder (Liu et al., 2021), and generalized anxiety disorder. These studies have demonstrated that analyzing dynamic regional brain activity can provide insight into the underlying neuropathologic mechanisms. However, few studies have analyzed changes in local intrinsic brain activity over time in patients with CI.

The aim of the present study was to investigate dynamic local brain activity in patients with CI. Using a sliding window approach, voxel-wise dynamic fALFF (dfALFF) maps were calculated and compared between patients with CI and healthy controls (HCs). Multivariate pattern analysis (MVPA) was conducted using dfALFF values of brain regions that differed between the two groups. We hypothesized that patients with CI would show altered dfALFF patterns that could be used to distinguish them from HC subjects.

## Materials and methods

### Study subjects

A total of 30 patients with CI were recruited at Ankang Hospital of Traditional Chinese Medicine (Shaanxi, China) from October 2020 to March 2022, and 27 HCs were recruited from the local community. The inclusion criteria for patients with CI were as follows: (1) Met the DSM-V criteria for CI; (2) had experienced difficulties in falling asleep or maintaining sleep, or early awakening for at least 3 months; (3) aged 25–65 years; (4) had no other sleep problems such as parasomnia or restless leg syndrome; (5) free of any psychoactive medication for at least 2 weeks prior to and during study participation; (6) insomnia was not caused by other organic diseases or serious mental diseases such as generalized anxiety disorder; and (7) right-handed. Exclusion criteria were as follows: (1) Medical history of severe organic brain disorders or brain surgery; (2) pregnancy or breastfeeding; (3) contraindications to MRI scans; (4) history of alcohol dependence or substance abuse; (5) abnormal conventional brain MRI findings such as tumors or subdural hematomas.

Healthy controls were recruited from the community and matched with CI patients on age, sex, and education level. Inclusion criteria for HCs were as follows: (1) Good sleep quality; (2) regular sleep habits; (3) no history of substance abuse or neurologic or psychiatric disorders; and (4) no abnormalities in conventional brain imaging examination.

The Ankang Hospital of Traditional Chinese Medicine Ethics Committee reviewed and approved this research. All subjects gave written, informed consent before participating in the study.

### Assessment of clinical indices

Each subject underwent a clinical characteristic assessment during recruitment. We used Pittsburgh Sleep Quality Index (PSQI) to evaluate subjects’ degree of insomnia; Hamilton Depression Rating Scale (HAMD) and Hamilton Anxiety Rating Scale (HAMA) to evaluate depression and anxiety, respectively; Fatigue Severity Scale (FSS) to assess fatigue; and Mini-Mental State Examination (MMSE) to assess general cognitive function.

### Magnetic resonance imaging acquisition

Cranial scans were performed on all subjects using an Ingenia CX 3.0-Tesla MR scanner (Philips, Amsterdam, The Netherlands) equipped with a 32-channel head coil. Earplugs and a foam pad were applied to dampen the noise of the machine and minimize head movement. Subjects were asked to remain stationary and not to fall asleep. Images with excessive head movement were excluded after scanning. The scan was performed by a trained technician. Routine MRI sequences were first obtained for each subject to exclude organic brain lesions.

An echo-plane imaging sequence was used for resting-state functional imaging, with the following scanning parameters: repetition time (*TR*) = 2,000 ms; echo time (*TE*) = 30 ms; 48 axial slices; slice thickness = 3.0 mm with no gap between slices; flip angle (*FA*) = 90°; matrix = 80 × 80; field of view (FOV) = 240 mm × 240 mm; voxel size = 3 mm × 3 mm × 3 mm; and 200 volumes.

Three-dimensional T1-weighted imaging data were acquired using a magnetization-prepared rapid gradient-echo sequence with the following parameters: *TR* = 6 ms; *TE* = 3.27 ms; flip angle = 8°; number of coronal slices = 190; slice thickness = 1.0 mm; FOV = 256 mm × 256 mm; and matrix = 256 × 256.

### Data preprocessing

Resting-state fMRI data were preprocessed with the Data Processing & Analysis of Brain Imaging toolkit (DPABI_V6.1) (Yan et al., 2016) based on Statistical Parametric Mapping 12.^1^ The first 10 time points were removed to eliminate the unstable magnetization effect. The remaining 190 volumes were corrected for slice-time delay. All other volumes were realigned to the middle volume to correct for head movement. Head movement parameters of all subjects were calculated by estimating the shift in each direction and angular rotation about each axis for each volume. Any subjects with head movement that exceeded 3.0 mm or 3.0° of axial rotation were excluded. Framewise displacement (FD), which measures changes in head position from volume to volume, was also computed. Each subject’s high-resolution T1-weighted images were coregistered to functional images. T1-weighted structural MR images were segmented into gray matter (GM), white matter (WM), and cerebrospinal fluid (CSF). These functional images were spatially normalized to standard Montreal Neurological Institute space. Several sources of nuisance signal including Friston-24 head movement parameters and average blood oxygenation level-dependent (BOLD) signals of the CSF and WM were regressed out from the time series. BOLD fMRI signal of each voxel were linearly detrended.

### Calculation of dynamic fractional amplitude of low-frequency fluctuation

The dfALFF was calculated by temporal dynamic analysis, which is incorporated in the DPABI sliding time window analysis.^2^ Window lengths used in previous studies ranged from 10 s (Thompson et al., 2013) to 180 s (Gonzalez-Castillo et al., 2015). A window length of 30–60 s was shown to be sufficient to detect changes in dynamic functional connectivity (Díez-Cirarda et al., 2018; Premi et al., 2019). There is currently no consensus on the optimal window length. In the present study, a window length of 30TR (60 s) and step size of 1TR (2 s) were applied to determine dfALFF for each subject according to the previous studies (Xue et al., 2020). The 190 time points were divided into 161 windows and the hamming window was used. The fALFF map within each window was computed, and the coefficient of variation (CV) of the fALFF at each voxel was calculated in all windows to assess the variability of fALFF. For standardization, CV maps were transformed into z-scores by subtracting the mean and dividing by the standard deviation of global values. Normalized images were spatially smoothed with an isotropic 4-mm full-width at half-maximum Gaussian kernel.

### Statistical analysis

We compared the demographic and clinical characteristics of the CI and HC groups with the chi-squared test, Mann–Whitney *U*-test, and 2-sample *t*-test using SPSS v25.0 software (IBM, Armonk, NY, USA). Intergroup differences in dfALFF were compared with the 2-sample *t*-test, with mean FD, age, sex, and education level as covariates. Multiple comparison correction was performed for the 2-sample *t*-test using a voxel-wise Gaussian random field (GRF) approach (single voxel, *p* < 0.001; cluster level, *p* < 0.05). To further investigate the relationship between the severity of symptoms in patients with CI and abnormal dfALFF variability, Pearson correlation analysis was performed for normally distributed data and Spearman correlation analysis was used for non-normally distributed data. Differences with *p* < 0.05 were considered statistically significant.

### Multivariate pattern analysis

Region of interest-wise MVPA was performed to assess the utility of abnormal dfALFF for distinguishing between patients with CI and HCs. Mean dfALFF values in brain regions with significant intergroup differences were extracted for each subject and used as input features in the models. The logistic regression classifier in the sklearn library (Pedregosa et al., 2011) for Python was used to train the models. The logistic regression model parameters were that penalty was L2 and C as the inverse regularization strength was one. Ten-fold cross-validation was used to evaluate the performance of our classifiers. The accuracy, sensitivity, and specificity were calculated to assess the classification result.

### Validation analysis

To further evaluate the accuracy and reliability of our results, different window lengths (20TR, 50TR), step size (3TR), and a 6-mm Gaussian smoothing kernel were applied and intergroup comparisons were repeated. There is at present no consensus on whether global signal regression should be applied in the preprocessing of resting-state fMRI data (Murphy and Fox, 2017). Another validation analysis was conducted to ensure that functional images were corrected by global signal regression.

## Results

### Demographic and clinical characteristics of the study population

There were no significant differences in age, sex, education level, FD score, and MMSE score between CI and HC groups. However, PSQI, HAMD, HAMA, and FSS scores were significantly higher in CI patients than in HCs (*p* < 0.05; Table 1).

**TABLE 1:** Demographic and clinical information of subjects.

Characteristics	Control (*n* = 27)	Insomnia (*n* = 30)	χ^2/^*t*/*z*	*P*-values
**Demographic data**				
Gender (male/female)	7/20	9/21	0.117	0.733^a^
Age (years)	49 ± 12	51 ± 13	–0.402	0.689^c^
Education level (years)	12 (7)	15 (5)	–1.642	0.101^b^
**Clinical scales**				
PSQI	5 (0)	16 (3)	–6.307	< 0.001^b^^*^
HAMD	7 (2)	23 (16)	–6.092	< 0.001^b^^*^
HAMA	4 (3)	8 (17)	–4.059	< 0.001^b^^*^
FSS	22 (1)	22 (3)	–2.245	< 0.001^b^^*^
MMSE	29 (2)	30 (2)	−1.090*CPSTABLEENTER*	0.276^b^
**Head motion parameter**				
Mean FD	0.08 (0.09)	0.09 (0.07)	–0.160	0.873^b^

FD, framewise displacement; PSQI, Pittsburgh Sleep Quality Index; HAMD, Hamilton Depression Rating Scale; HAMA, Hamilton Anxiety Rating Scale; FSS, Fatigue Severity Scale; MMSE, Mini-Mental State Examination.

^a^Chi-square test; ^b^Mann–Whitney *U*-test; ^c^2-sample *t*-test (two-tailed).

*p < 0.05.

### Changes in dynamic fractional amplitude of low-frequency fluctuation in patients with chronic insomnia

**FIGURE 1:**
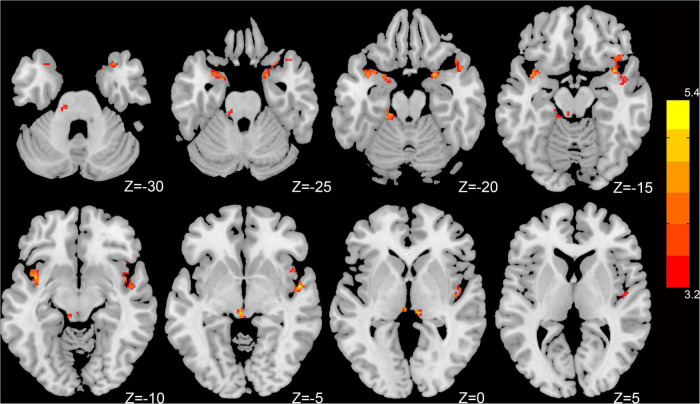
Dynamic intrinsic brain activity with significant intergroup differences in dfALFF between the CI group and HC group. Cluster-level *p* < 0.05, GRF corrected. Red colors indicate significantly increased dfALFF in CI patients. The right color bar indicates *t*-values from a global cluster-based 2-sample *t*-test analysis.

Compared to HCs, patients with CI had significantly increased dfALFF in the left insula, right superior temporal gyrus (STG), left parahippocampal gyrus, right amygdala, and bilateral cerebellum_9 (belonging to cerebellum posterior lobe) (*p* < 0.05, GRF-corrected; Table 2 and Figure 1).

**TABLE 2:** Regions with changed dfALFF between patients with CI and HCs.

Brain regions		L/R	Cluster size (voxels)	Peak MNI coordinates			*t*-values
AAL	Brodmann			*x*	*y*	*z*	
**CI>HC**							
Insula	BA48	L	195	–39.5	5.5	–12.5	5.08
Superior temporal gyrus	BA48	R	230	50.5	–2.5	–4.5	5.46
Parahippocampal	BA30	L	81	–17.5	–28.5	–18.5	5.20
Amygdala	BA34	R	83	24.5	5.5	–18.5	4.89
Cerebelum_9	/	L	176	–13.5	–44.5	–48.5	5.11
Cerebelum_9	/	R	88	14.5	–34.5	–44.5	4.69

AAL, anatomical automatic labeling; MNI, Montreal Neurological Institute; L, left hemisphere; R, right hemisphere; dfALFF, the dynamic fractional amplitude of low-frequency fluctuations; CI, chronic insomnia; HC, healthy control.

### Correlational analysis

**FIGURE 2:**
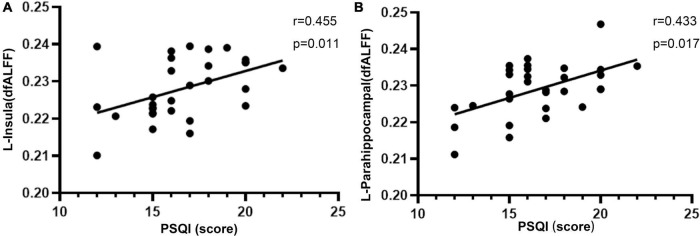
Significant positive correlations between the altered dfALFF in the left insula **(A)**, left parahippocampal **(B)** and PSQI in CI patients. dfALFF, the dynamic fractional amplitude of low-frequency fluctuation; CI, chronic insomnia; PSQI, Pittsburgh Sleep Quality Index.

There was a significant positive correlation between PSQI score and dfALFF in the left insula (*r* = 0.455, *p* = 0.011) and left parahippocampal gyrus (*r* = 0.433, *p* = 0.017; Figure 2).

### Classification results

We established a logistic regression model that had a sensitivity of 96.7%, specificity of 80.0%, and overall accuracy of 83.0% for distinguishing between patients with CI and HC subjects.

### Validation results

We used different sliding window lengths and step size to validate our primary MRI findings (Supplementary material). The results obtained with sliding window lengths of 20TR (Supplementary Figure 1) and 50TR (Supplementary Figure 2) were similar to those obtained with 30TR. Meanwhile, a sliding window step size of 3TR (Supplementary Figure 3) yielded a result similar to 1TR. Additionally, by using a 6-mm Gaussian kernel to smooth dfALFF maps, the result was similar to using a 4-mm Gaussian kernel (Supplementary Figure 4). The result obtained after global signal regression correction was consistent with the main result without correction (Supplementary Figure 5).

## Discussion

The present study used a dfALFF method to investigate the temporal variability of local brain activity in CI. Compared with HC subjects, patients with CI showed higher variability in dfALFF in the left insula, right STG, part of the subcortical nucleus, and bilateral cerebellum posterior lobes. This distinguished CI patients from HCs with an accuracy of 83%, and the variability in the left insula and left parahippocampal gyrus were positively associated with symptom severity in CI. These results imply that the neural mechanisms of CI may involve pathologic changes in dynamic regional brain activity.

In the present study, patients with CI showed greater dfALFF variability in the left insula compared with HCs. The insula is a central hub in the salience network (Menon, 2011). Using simultaneous EEG and fMRI, it was demonstrated that patients with insomnia had aberrant activation in the anterior insula of the salience network (Chen et al., 2014); and a positron emission tomography-computed tomography study found that an increase in the relative rate of glucose metabolism in the right anterior insula during non-rapid eye movement sleep was related to abnormal sleep onset latency in patients with insomnia (Kay et al., 2017). Meanwhile, cognitive arousal in patients with insomnia stimulated the hyperarousal network during sleep and was associated with longer sleeping latencies, as observed by 24-h polysomnography (PSG) monitoring (Kalmbach et al., 2020). In our study, abnormal dfALFF variability in the insula was associated with reduced sleep quality, suggesting that it can be a potential biological marker that reflects the severity of insomnia.

The amygdala is part of the limbic system and an important subcortical region in the salience network (Menon, 2011) that plays a key role in emotion processing (Sah, 2017). An fMRI study found increased activation in the amygdala in response to negative insomnia-related emotional stimuli in insomnia (Baglioni et al., 2014), and brain morphometry studies have revealed atrophy of amygdala GM in patients with CI (Koo et al., 2017; Emamian et al., 2021). Regional atrophy in the amygdala has been linked to abnormalities in emotional behavior (Gong et al., 2019). Additionally, changes in functional connectivity in the amygdala has been observed in insomnia patients (Huang et al., 2012; Pace-Schott et al., 2017). Thus, abnormal variability in local activity in the insula and amygdala may reflect involvement of the salience network in the pathophysiology of insomnia.

A recent neuroimaging study of 1,074 adults found a correlation between sleep quality and hippocampus and parahippocampal gyrus GM volume in female subjects (Neumann et al., 2020). The results of a meta-analysis of 28 studies showed that higher activity in the parahippocampal gyrus was related to dysfunctional emotion regulation (Jiang et al., 2020). In the present work, aberrant activity in the parahippocampal gyrus was associated with poor sleep quality, but was unrelated to emotion scores. The discrepancy between our results and previous observations may be attributable to differences in the study population and methodology. The functional significance of abnormal activity in the parahippocampal gyrus in CI warrants further examination.

Bilateral posterior lobes of the cerebellum showed increased dfALFF variability in CI patients. The cerebellum not only controls motor function (Manto and Oulad Ben Taib, 2013) but also regulates sleep (DelRosso and Hoque, 2014). It was reported that CI patients had a significant decrease in static ALFF in bilateral cerebellum compared with normal subjects (Li et al., 2016). Moreover, cerebellum GM volume was reduced in CI patients (Joo et al., 2013). These findings provide evidence that abnormalities in the cerebellum contribute to sleep dysregulation.

In clinical practice, insomnia is primarily diagnosed based on self-reported symptoms that can be mistaken for symptoms of other diseases (Benjamins et al., 2017). Therefore, it is necessary to develop other objective and reliable approaches which could be used to assist in the diagnosis of patients with CI. The logistic regression model generated in the current work based on differences in dfALFF in various brain regions between CI patients and HC subjects had a relatively high accuracy (83%) for distinguishing between the two groups. This is consistent with the results of previous studies that used MVPA to differentiate patients with CI from HCs (Dai et al., 2020; Gong et al., 2022), and suggests that dfALFF is a potential biomarker for the objective diagnosis of CI.

There were several limitations to the present study that should be acknowledged. First, the small size of study population limited the accuracy of our model. Second, existing scales used to evaluate sleep quality are relatively subjective. Third, the cross-sectional study design precluded an exploration of the causal relationship between altered brain activity and clinical indices. Therefore, further research is needed with a larger sample; and using methods such as PSG and sleep actigraphy can also allow the objective assessment of sleep quality.

## Conclusion

The results of this study showed that there was increased instability in brain activity in multiple brain regions of patients with CI. Additionally, dynamic regional neural activity in the insula was associated with insomnia symptoms. Thus, dynamic regional functional measurements may help to clarify the underlying neurobiologic mechanisms of CI.

## Data availability statement

The raw data supporting the conclusions of this article will be made available by the authors, without undue reservation.

## Ethics statement

The studies involving human participants were reviewed and approved by the Ankang Hospital of Traditional Chinese Medicine Ethics Committee. The patients/participants provided their written informed consent to participate in this study. Written informed consent was obtained from the individual(s) for the publication of any potentially identifiable images or data included in this article.

## Author contributions

XH and WC designed the experiments. WC, HW, TS, and QW performed the experiments. WC and HW analyzed the all data. HW, WH, QL, YL, and YZ wrote and revised the manuscript. All authors approved the final manuscript.

## Footnotes

1
www.fil.ion.ucl.ac.uk/spm/

2
http://rfmri.org/DPABI
