# Supplementary material for: Dynamic changes in fractional amplitude of low-frequency fluctuations in patients with chronic insomnia
Source: Front Neurosci. 2022 Nov 29;16:1050240. doi: 10.3389/fnins.2022.1050240 (PMC9744813; doi:10.3389/fnins.2022.1050240)
Supplement: Supplementary file 1 [file Table_1.DOC]

Supplementary Material

We applied 30TR as window length, 1TR as step size, 4mm as smoothing core in our main research. To test if the different window length, step size, Gaussian smooth kernel, and global signal regression have effects on our results, we tried other parameter, and the figure below demonstrated the stability of our experiments.

1. **Results from different window length**
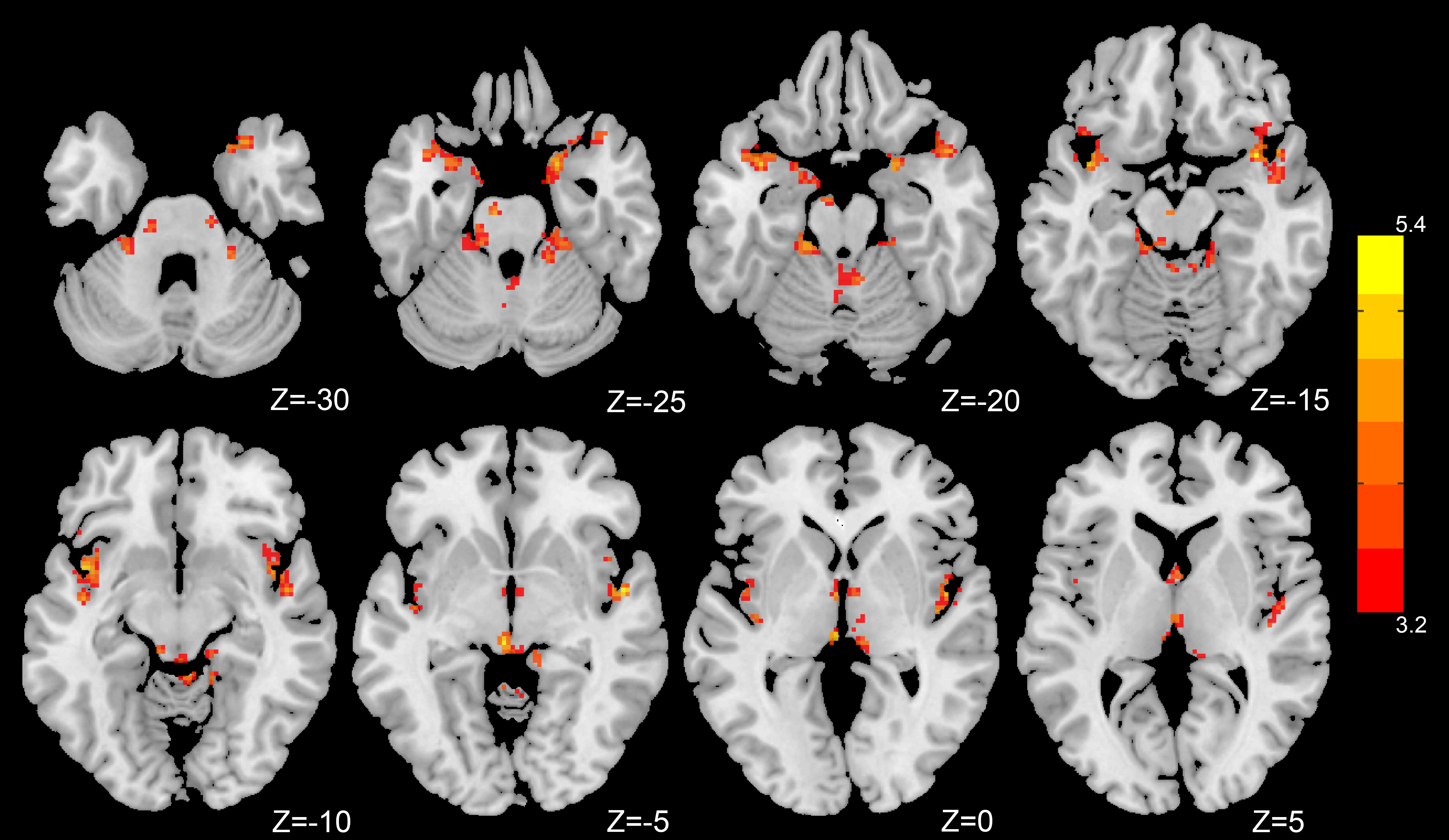


**Supplementary Figure 1.** Window length, 20 TR; Window step size, 1 TR; Gaussian smooth kernel, 4mm.


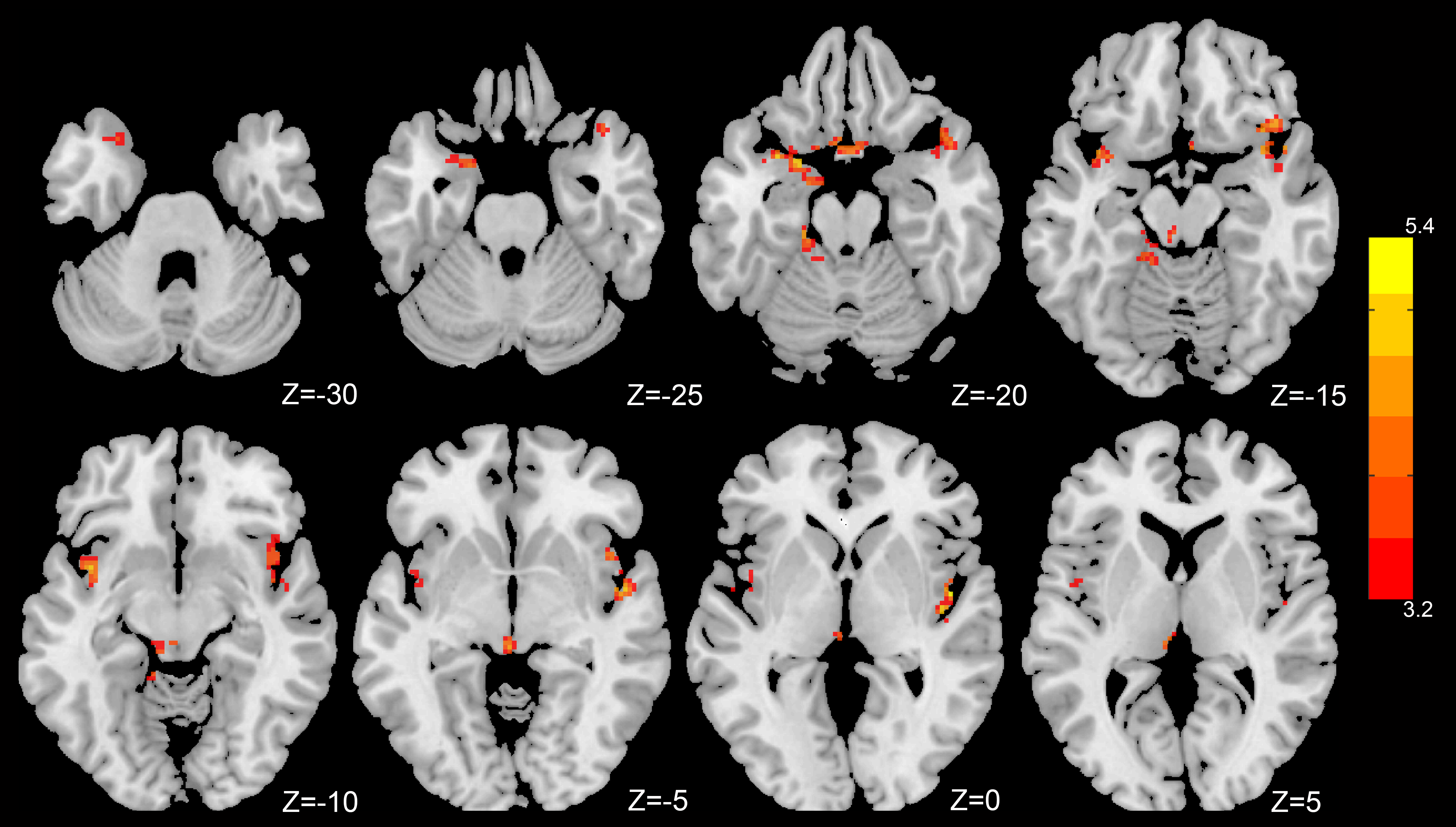


**Supplementary Figure 2.** Window length, 50 TR; Window step size, 1 TR; Gaussian smooth kernel, 4mm.

1. **Results from different window step size**


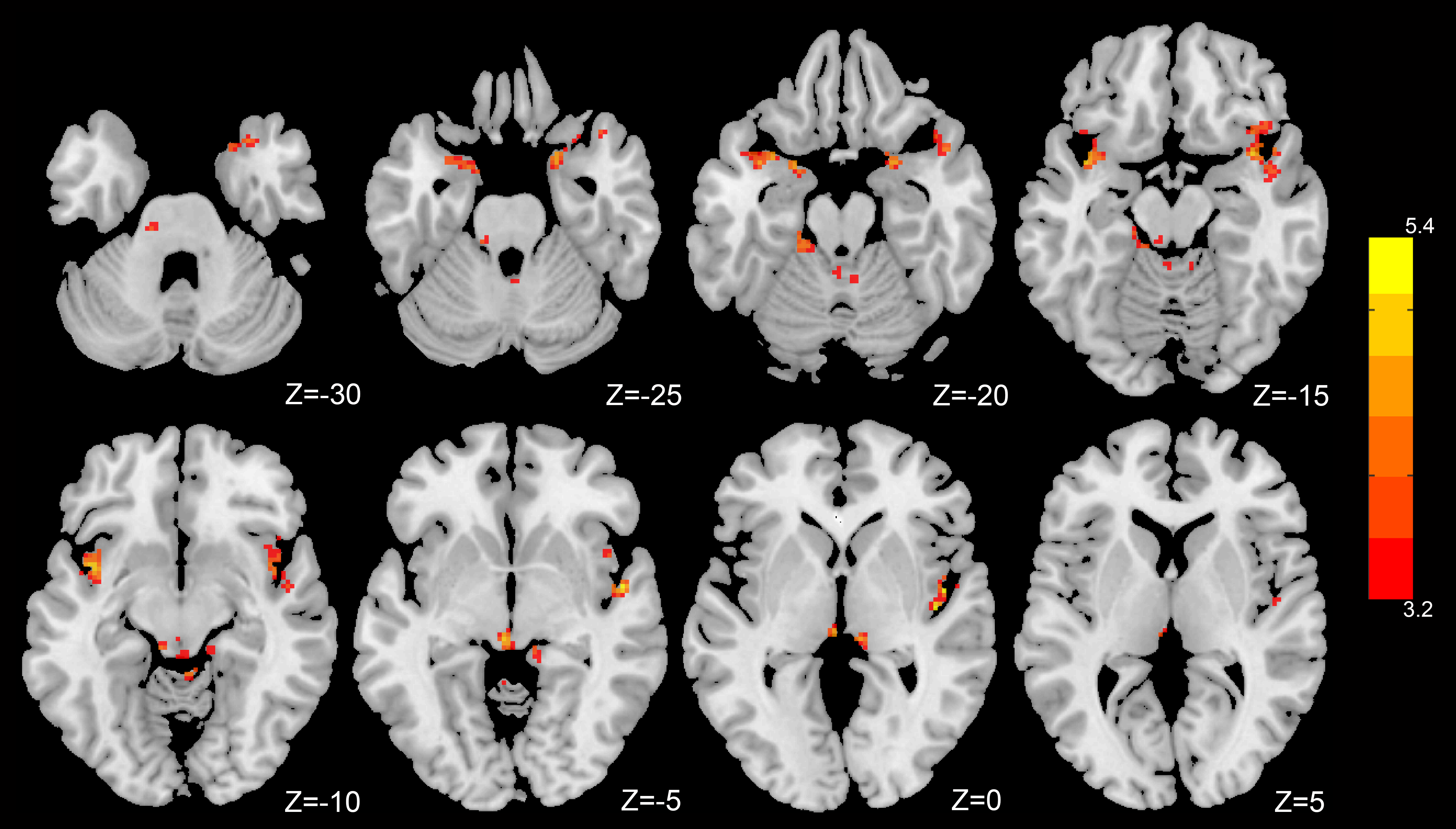


**Supplementary Figure 3.** Window length, 30 TR; Window step size, 3 TR; Gaussian smooth kernel, 4mm.

1. **Results from different Gaussian core**


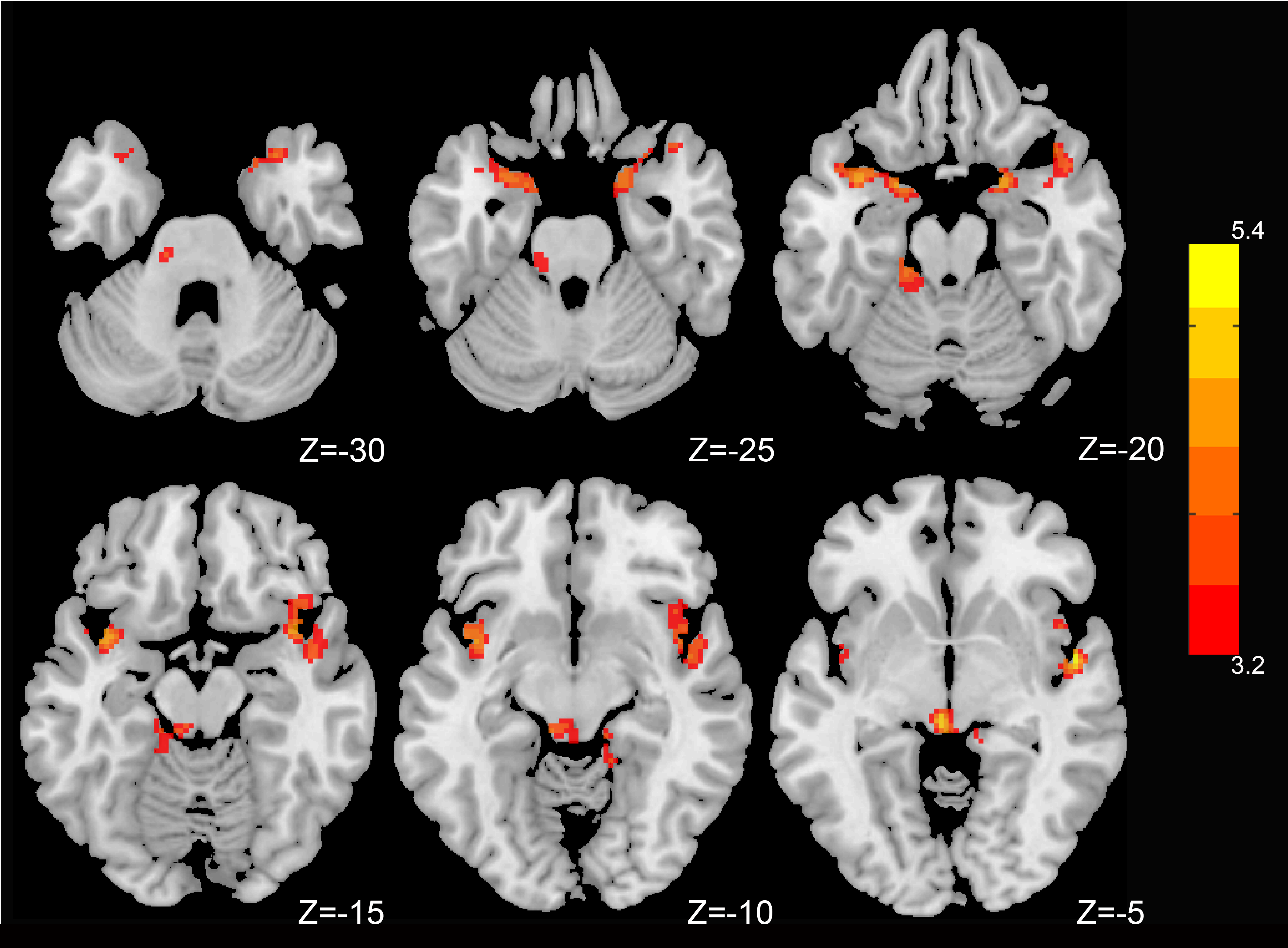


Supplementary Figure 4. Window length, 30 TR; Window step size, 1TR; Gaussian smooth kernel, 6mm.

1. **Results after regressing the global signal in the preprocessing**


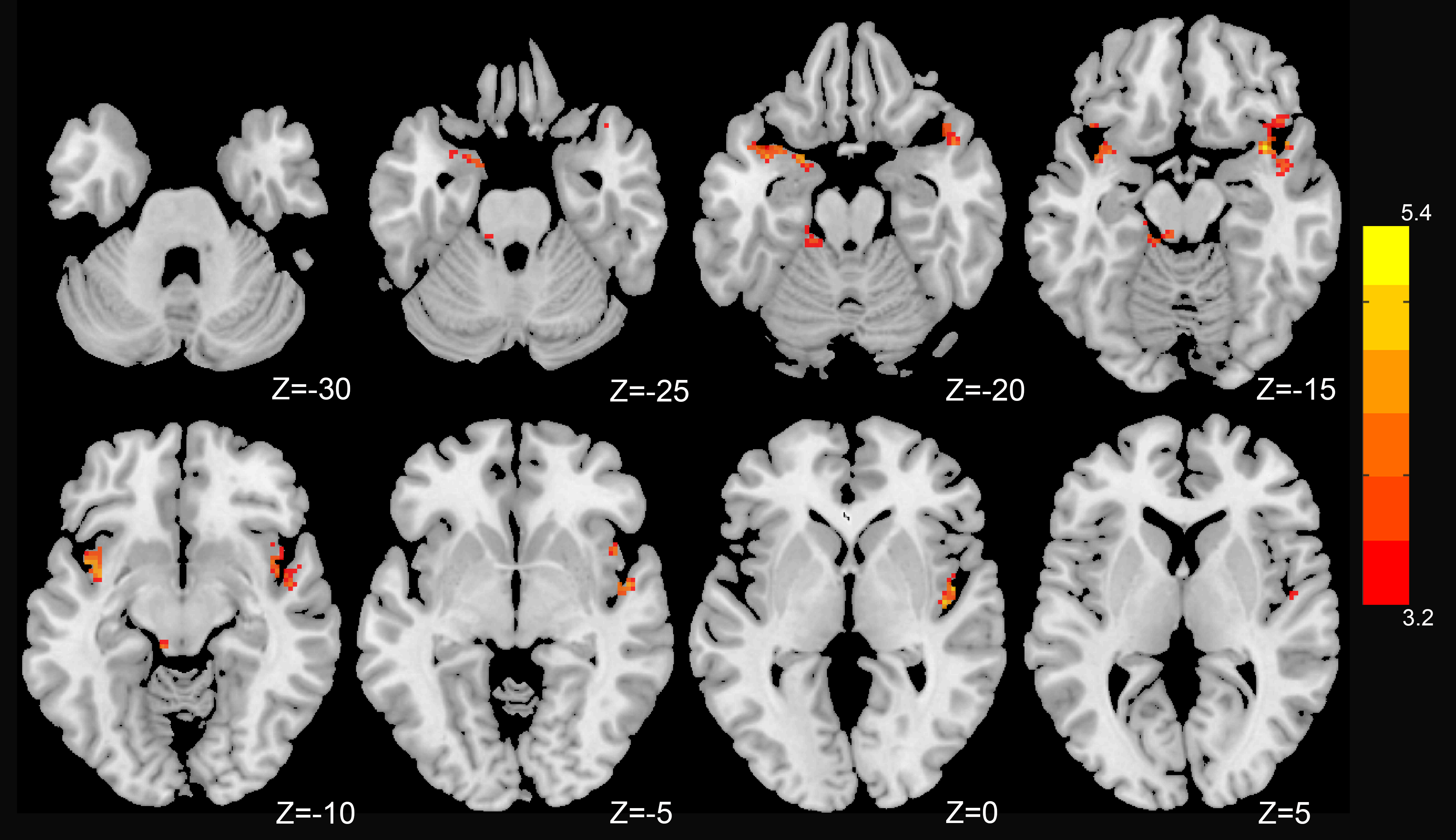


**Supplementary Figure 5.** Window length, 30 TR; Window step size, 1TR; Gaussian smooth kernel, 4mm; regression to the global signal.
